# Electrocardiographic Abnormalities in Elderly Patients Receiving Psychotropic Therapy in the Emergency Department: A Retrospective Cohort Study

**DOI:** 10.3390/life15101545

**Published:** 2025-10-01

**Authors:** Marianna Mazza, Marcello Covino, Filippo Bambini, Enrico Romagnoli, Giuseppe Biondi-Zoccai, Mariella Fuorlo, Fabiana Barone, Simona Racco, Benedetta Simeoni, Francesco Franceschi, Gabriele Sani, Giuseppe Marano

**Affiliations:** 1 Unit of Psychiatry, Fondazione Policlinico Universitario Agostino Gemelli IRCCS, 00168 Rome, Italy; 2 Department of Neurosciences, Università Cattolica del Sacro Cuore, 00168 Rome, Italy; 3 Emergency Medicine Department, Fondazione Policlinico Universitario Agostino Gemelli IRCCS, Università Cattolica del Sacro Cuore, 00168 Rome, Italy; 4 Department of Cardiovascular Sciences, Fondazione Policlinico Universitario Agostino Gemelli IRCCS, 00168 Rome, Italy; 5 Department of Medical Surgical Sciences and Biotechnologies, Sapienza University of Rome, 04100 Latina, Italy; 6 GVM Care & Research, Maria Cecilia Hospital, 48033 Cotignola, Italy

**Keywords:** QTc prolongation, electrocardiography, psychotropic drugs, elderly, emergency department, cardiac safety, polypharmacy, antipsychotics, antidepressants, geriatric psychiatry

## Abstract

Background: Psychotropic medications are frequently prescribed to elderly patients in emergency settings, yet their potential to induce electrocardiographic (ECG) abnormalities, particularly QTc interval prolongation, raises safety concerns. Older adults may be especially vulnerable due to polypharmacy, age-related cardiac changes, and comorbidities. Methods: We conducted a retrospective observational study on patients aged ≥65 years who underwent psychiatric evaluation in the Emergency Department (ED) of a tertiary hospital between 2015 and 2023. Data was extracted on demographics, psychiatric symptoms, psychotropic drug use, and ECG findings. The primary outcome was the prevalence of major ECG abnormalities (QTc or QRS prolongation), and secondary analyses explored associations with drug class and hospitalization. Results: Seventy-seven patients were included (62.3% female, median age 74 years). Overall, 22.1% exhibited ECG abnormalities, with QTc prolongation in 16.9% and QRS widening in 5.2%. ECG alterations were more common among patients receiving psychotropic drugs (30.7% vs. 13.2%; *p* = 0.046). Multivariate analysis confirmed psychotropic therapy as an independent predictor of ECG abnormalities (OR 2.84; 95% CI: 1.01–7.98; *p* = 0.049). No significant sex-related differences were observed. Conclusions: ECG abnormalities are common in elderly patients undergoing psychiatric assessment in the ED and seem associated with psychotropic medication use. However, non-pharmacological factors also contribute significantly to risk. Integrated multidisciplinary evaluation is essential to ensure both psychiatric and cardiovascular safety in this fragile population.

## 1. Introduction

Psychotropic medications, including antipsychotics, antidepressants, and mood stabilizers, are widely used in elderly populations for the management of psychiatric conditions such as depression, agitation, and psychosis [1]. These medications are not devoid of cardiovascular risk, particularly concerning their potential to cause electrocardiographic (ECG) abnormalities [2,3]. Among these, prolongation of the QT interval (time from the start of the Q wave to the end of the T wave) and widening of the QRS complex (defined as a large deflection in the ECG that represents the almost synchronous depolarization of the ventricles, lasting 0.1 s or less) are particularly relevant due to their association with life-threatening arrhythmias, such as torsades de pointes (TdP) and sudden cardiac death (SCD) [4]. While the arrhythmogenic potential of psychotropic drugs has been well documented in younger and middle-aged adults, elderly patients represent a uniquely vulnerable subgroup [5,6].

Age-related changes in cardiac electrophysiology, higher prevalence of comorbidities (e.g., coronary artery disease, electrolyte imbalances), polypharmacy, and altered pharmacokinetics contribute to a significantly increased risk of adverse cardiac events [7]. In fact, those older than age 70 are 3.5 times more likely than younger individuals to be admitted to the hospital due to adverse drug reactions associated with psychotropic medications. The risk for adverse reactions increases dramatically with the number of medications used and with increasing age [8,9]. Despite this, elderly patients are underrepresented in clinical trials and there is a relative paucity of real-world data assessing the burden and risk profile of ECG abnormalities in this population, particularly in acute care settings such as emergency departments (EDs) [10,11,12].

This study aims to address this gap by retrospectively evaluating the prevalence of clinically significant ECG alterations in elderly patients (>65 years) receiving psychotropic medications and referred for psychiatric evaluation in the ED. We adopted an age cut-off of ≥65 years, which is a widely accepted threshold to define older adults in both geriatric medicine and psychiatric epidemiology. This criterion also facilitates comparability with most prior studies investigating psychotropic safety in elderly populations [10,11,12].

Specifically, the study focuses on QT interval prolongation and QRS widening, which have been previously linked to the use of antipsychotics, selective serotonin reuptake inhibitors (SSRIs), serotonin-norepinephrine reuptake inhibitors (SNRIs), Serotonin Antagonist and Reuptake Inhibitors (SARIs) and other psychotropic agents [13,14]. By identifying the prevalence and risk factors associated with these ECG changes, the study seeks to inform risk stratification strategies and safer pharmacologic management in geriatric emergency psychiatry. We specifically focused on patients undergoing psychiatric consultation in the ED, as this subgroup represents a clinically relevant interface where psychotropic therapy is often initiated or adjusted, and where collaboration between psychiatry and emergency medicine is crucial.

## 2. Materials and Methods

### 2.1. Study Design and Setting

This retrospective observational study was conducted at the Fondazione Policlinico Universitario A. Gemelli IRCCS (Rome, Italy), a tertiary academic hospital whose Emergency Department (ED) handles approximately 80,000 adult patient visits annually. The study received approval from the Fondazione Policlinico Agostino Gemelli Protocol N°: 0025817/22, ID: 5121 of 3 August 2022.

### 2.2. Study Population

We reviewed the electronic medical records of all patients aged ≥65 years who presented to the ED and underwent a psychiatric consultation between 1 January 2015, and 31 December 2023. Patients with incomplete ECG data or missing documentation on psychiatric medications were excluded. The relatively small number of cases reflects the highly selective nature of our study population: elderly patients (≥65 years) who underwent both psychiatric consultation and 12-lead ECG in the ED, with complete documentation of psychotropic drug use. Patients with incomplete or missing ECG or medication records were excluded, which substantially reduced the final sample size. Although no a priori sample size calculation was feasible due to the retrospective design, a post-hoc power analysis indicated that the study had approximately 70% power to detect an odds ratio >2.5 for ECG abnormalities.

### 2.3. Data Collection

The following data were extracted: demographics (age, sex); clinical presentation (vital signs at triage), symptoms (e.g., agitation, anxiety, altered mental status, chest pain, syncope, vertigo), inappropriate drug intake, trauma; medical history (comorbidities including hypertension, cardiovascular disease, and diabetes); ECG findings. Patients with a documented diagnosis of congenital long QT syndrome were excluded. Because of the retrospective design, systematic access to baseline ECGs prior to ED presentation was not available, and therefore we could not reliably exclude pre-existing QTc prolongation unrelated to psychotropic exposure.

All patients underwent 12-lead ECGs interpreted by senior emergency physicians and abnormalities were classified as: QTc prolongation: QTc > 450 ms in males or >470 ms in females; QRS prolongation: QRS ≥ 120 ms; psychotropic medications: administration of antidepressants (SSRIs, SNRIs, SARIs), antipsychotics (typical and atypical), benzodiazepines, lithium, and anticonvulsant mood stabilizers (e.g., valproic acid). Thresholds for QTc prolongation are consistent with published guidelines and prior systematic reviews on drug-induced QTc prolongation [15]. QTc intervals were calculated using Bazett’s correction formula, in accordance with the ED’s standard ECG reporting system.

Information on exact dose ranges, treatment duration, and timing of psychotropic initiation relative to ED admission was not consistently available in the retrospective dataset. ECGs were systematically performed at ED presentation, regardless of whether patients were chronic users or recent initiators of psychotropic therapy.

### 2.4. Outcomes

The primary outcome was the prevalence of major electrocardiographic abnormalities (QTc or QRS prolongation) among elderly ED patients undergoing psychiatric evaluation. Secondary analyses investigated associations between psychotropic drug use and ECG abnormalities, as well as differences based on sex, hospitalization, and psychiatric treatment.

### 2.5. Statistical Analysis

Categorical variables are presented as frequencies and percentages and compared using the chi-square test with Yates correction or Fisher’s exact test, as appropriate. Continuous variables are reported as medians with interquartile ranges (IQR) and compared using the Mann–Whitney U test. Univariate and multivariate logistic regression analyses were performed to identify independent predictors of ECG abnormalities. A two-sided *p*-value < 0.05 was considered statistically significant. Analyses were performed using the SPSS v26^®^ (IBM, Armonk, NY, USA, 2018).

## 3. Results

A total of 77 patients aged ≥65 years (mean age 74 years, range 66–88) who underwent psychiatric evaluation in the Emergency Department were included in the study. The majority were female (*n* = 48, 62.3%), while 29 were male (37.7%). Table 1 reports the clinical presentation, triage classification, and therapeutic features of elderly patients undergoing psychiatric evaluation in the Emergency Department, stratified by sex.

**Figure 1:**
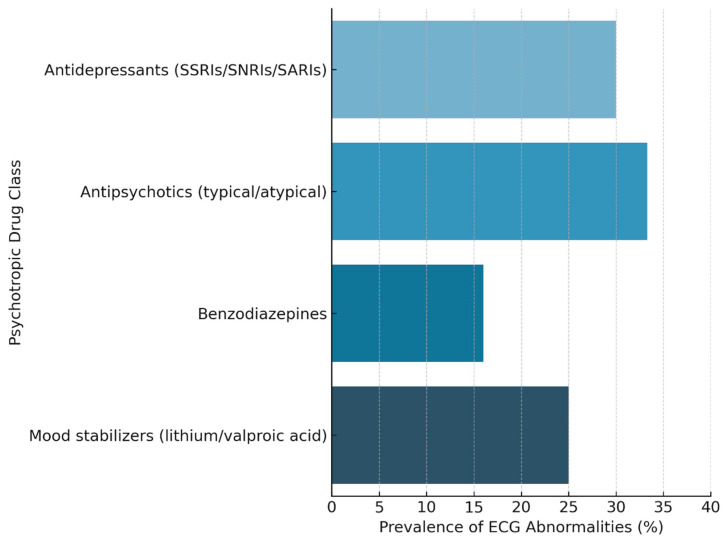
Prevalence of ECG Abnormalities by Psychotropic Drug Class. Note: Electrocardiographic abnormalities include QTc interval prolongation (QTc > 450 ms in males, >470 ms in females) and/or QRS complex widening (QRS ≥ 120 ms). Percentages are calculated within each drug class subgroup. Data derived from a retrospective analysis of elderly patients undergoing psychiatric evaluation in the Emergency Department. Abbreviations: SSRIs-Selective Serotonin Reuptake Inhibitors, SNRIs-Serotonin-Norepinephrine Reuptake Inhibitors, SARIs-Serotonin Antagonist and Reuptake Inhibitors.

**Figure 2:**
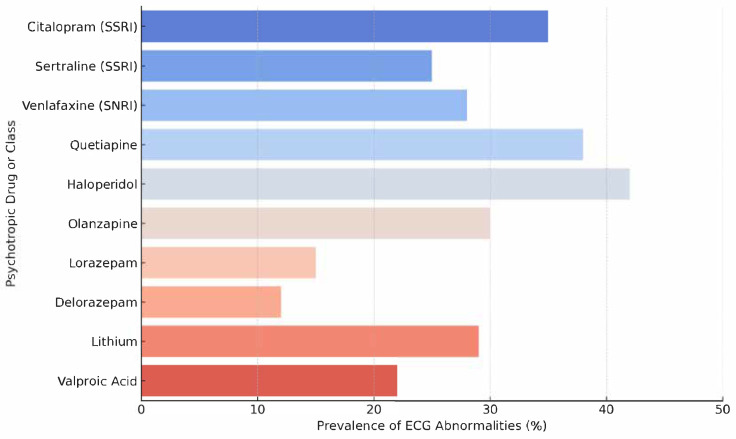
ECG Abnormality Prevalence by Specific Psychotropic Agent. Note: Data are based on observed clinical records and are representative of the most frequently prescribed drugs in this population. ECG abnormalities were defined as QTc > 450 ms in males or >470 ms in females, and/or QRS duration ≥ 120 ms. Percentages refer to the proportion of patients treated with each drug who exhibited at least one abnormality on ECG.

Overall, 39 patients (50.6%) were prescribed at least one psychotropic medication during their ED admission. The most frequently used drug classes included: antidepressants (SSRIs, SNRIs, SARIs), antipsychotics (quetiapine, haloperidol, olanzapine), benzodiazepines, mood stabilizers (lithium, valproic acid) (Figure 1). Among the entire cohort, 17 patients (22.1%) exhibited major ECG abnormalities, defined as either QTc prolongation or QRS complex widening. Overall, 17 patients (22.1%) exhibited major ECG abnormalities. QTc prolongation was observed in 13 patients (16.9%), while QRS prolongation (≥120 ms) was found in 4 patients (5.2%). Stratified analyses are reported in Table 2 and Figure 2.

Following ED evaluation, 41 patients (53.2%) were admitted to hospital. Hospitalized patients were more likely to be receiving psychotropic medications (*p* = 0.02), and a trend toward higher ECG abnormality prevalence was noted in this subgroup, though not statistically significant (*p* = 0.09). No statistically significant differences were found between male and female patients regarding ECG abnormalities (*p* = 0.62), psychiatric medication use (*p* = 0.87), or hospitalization rates (*p* = 0.41). In the multivariate logistic regression model, psychotropic drug therapy emerged as a suggestive independent predictor of ECG alterations (OR 2.84, 95% CI: 1.01–7.98, *p* = 0.049), after adjusting for age, sex, and cardiovascular comorbidities.

Table 2 presents the results of the multivariate logistic regression model assessing independent predictors of ECG abnormalities (QTc or QRS prolongation) in elderly patients undergoing psychiatric evaluation in the Emergency Department.

## 4. Discussion

In our cohort of elderly Emergency Department (ED) patients undergoing psychiatric evaluation, 22.1% exhibited major ECG abnormalities, primarily QTc prolongation (16.9%) and to a lesser extent QRS widening (5.2%). Importantly, QTc prolongation and QRS widening were analyzed separately, given their distinct mechanisms, prognostic implications, and management strategies. QTc prolongation was the predominant abnormality in our cohort, whereas QRS widening was less common.

Psychotropic drug use was significantly associated with ECG alterations (OR ≈ 2.8), even after adjusting for age, sex, and cardiovascular comorbidity. We emphasize that this association, while statistically significant, is based on a borderline *p*-value and a wide confidence interval, which indicates statistical fragility. Therefore, our findings should be interpreted with caution.

Although research in psychiatric populations suggests a prevalence of QTc prolongation of around 4% in antipsychotic-treated patients [16,17], our rate is markedly higher. This is in line with studies in elderly and comorbid populations, where age > 65 years, female sex, and structural heart disease amplify the risk of repolarization abnormalities [15,18]. Moreover, exposure to multiple QT-prolonging drugs has not consistently predicted QTc prolongation. Rather, non-pharmacological factors such as advanced age, electrolyte imbalances, and cardiac comorbidities are often stronger predictors in real-world gerontopsychiatric cohorts [4,19]. Our findings support this model: psychotropic therapy was associated with a higher likelihood of ECG abnormalities, but many affected patients also presented additional risk factors [20,21,22].

QTc prolongation results from delayed ventricular repolarisation, often via inhibition of the rapid component of the delayed rectifier potassium current, most notably via hERG channel blockade [23]. Most antipsychotics have QTc-prolonging potential, but agents such as ziprasidone and thioridazine carry the highest risk, whereas aripiprazole and lurasidone are considered safer [24,25,26,27,28].

Intravenous haloperidol has been associated with QTc prolongation and torsades de pointes (TdP), particularly in medically ill older adults [29,30].

Although antidepressants have traditionally been considered safer, citalopram has shown dose-dependent QTc prolongation, leading regulatory agencies to limit their use in older adults [31].

Escitalopram, despite structural similarity, appears to have a milder effect [32]. Fluoxetine, fluvoxamine, and sertraline at traditional doses demonstrate a lack of clinically significant increases in QTc [33]. In general, available data indicate that tricyclic antidepressants (TCAs) and citalopram pose the greatest risk for QT prolongation in older adults [34,35].

An analysis of ECG abnormality prevalence by specific psychotropic agent is shown in Figure 2, illustrating the heterogeneity in cardiotoxic potential across different pharmacological classes.

As shown in Figure 2, first-generation antipsychotics such as haloperidol demonstrated the highest rates of ECG abnormalities (42%), followed by second-generation agents like quetiapine (38%). These findings are consistent with previous pharmacodynamic studies indicating that antipsychotics can block the hERG potassium channel, prolonging ventricular repolarization and predisposing to QTc interval prolongation and, in extreme cases, torsades de pointes (1,6,9). Interestingly, even within the SSRI class, citalopram was associated with a higher prevalence of ECG abnormalities (35%) compared to sertraline (25%), reflecting its well-documented dose-dependent effect on QTc lengthening [34,36]. Benzodiazepines, such as lorazepam and delorazepam, exhibited relatively low rates of ECG abnormalities (<15%), in line with their minimal direct impact on cardiac conduction [37,38]. Mood stabilizers such as lithium and valproic acid presented intermediate prevalence values (22–29%), which may reflect their more indirect contributions to arrhythmogenic risk, such as through electrolyte disturbances or drug interactions [39,40]. It is worth noting that these findings, though informative, are drawn from a modestly sized and observational sample, and thus should be interpreted with caution.

Nonetheless, the figure supports the notion that not all psychotropics carry the same cardiac risk, and individualized risk–benefit evaluations should consider both the psychiatric indication and the specific electrophysiological profile of the agent in question [41].

This level of granularity may guide clinicians in selecting safer alternatives, especially in elderly patients with underlying cardiac vulnerabilities, while avoiding unnecessary withholding of essential psychiatric treatments [42,43].

Our data contribute to reinforce that elderly patients receiving psychotropic medications, especially those with pre-existing cardiac disease, require ECG monitoring. Given the low absolute change in QTc typical for individual drugs (often <20 ms), arrhythmic risk becomes clinically relevant only when multiple vulnerabilities converge. Rather than avoiding psychotropic medications altogether, clinicians should undertake a risk–benefit analysis, optimize modifiable risk factors (e.g., hypokalemia, hypomagnesemia), and prioritize drugs with lower QT liability when feasible [44,45].

This study presents several noteworthy strengths. Its real-world observational design reflects the complexity and heterogeneity of elderly patients accessing emergency psychiatric services, a population often excluded or underrepresented in clinical trials [46].

By focusing specifically on individuals aged 65 years and older, the study captures a clinically vulnerable subgroup at heightened risk for both psychotropic-related adverse events and baseline cardiovascular instability. The setting, an urban academic Emergency Department, enhances the ecological validity of the findings, as it mirrors the frontline challenges encountered by clinicians in managing acute neuropsychiatric presentations in older adults. The inclusion of detailed electrocardiographic data, interpreted in the context of psychiatric pharmacotherapy, offers a relevant intersection between cardiology and psychiatry, often overlooked in daily practice [47,48,49]. The use of multivariable analysis, although limited by sample size, allowed us to control for potential confounding variables such as age, sex, and pre-existing cardiovascular conditions. This analytical approach contributes to deepen our knowledge about the possible association between psychotropic drug use and ECG abnormalities. Although our results are consistent with prior studies, they provide real-world evidence specifically in the acute ED setting, a context where elderly patients are underrepresented and data remains scarce. A trend toward higher prevalence of ECG abnormalities was noted in hospitalized patients, although this observation should be considered exploratory and interpreted with caution. This study underscores the need for future prospective, multicenter, and mechanistic research.

However, this study is not without limitations. Most notably, its retrospective design implies an inherent risk of selection bias, incomplete data capture, and limited control over the timing and consistency of ECG assessments. Moreover, while our sample size (*n* = 77) is comparable to similar studies in this domain, it limits the statistical power to detect subtle associations or to conduct robust subgroup analyses, particularly for individual drug classes or combinations. The single-center nature of the study and the relatively small sample size limit the generalizability of our findings and reduce statistical power to detect subtle associations.

Another important limitation is the lack of longitudinal follow-up. Because the study is based solely on ED encounters, we cannot ascertain the persistence, reversibility, or clinical impact of the observed ECG abnormalities over time. For instance, it remains unclear whether QTc prolongation observed acutely resolved with medication adjustment or was sustained and associated with adverse outcomes such as arrhythmias or mortality.

Additionally, we did not systematically compare different QT correction formulas, which could influence the classification of borderline cases [50]. Another limitation is that baseline ECGs prior to ED presentation were not systematically available, and we cannot fully exclude the possibility that some QTc abnormalities were pre-existing rather than drug-related.

Finally, laboratory data on electrolytes, renal function, and serum drug levels were not uniformly available, which precluded a more granular risk stratification. We lacked systematic information on psychotropic dose, duration of treatment, and timing of ECG acquisition relative to drug exposure, which limits the ability to assess dose-response relationships. Laboratory parameters such as serum electrolytes, renal function, and psychotropic serum levels were not consistently available, which may have biased the observed associations and limited more granular risk stratification. Data on concomitant non-psychotropic QT-prolonging medications (e.g., macrolide antibiotics, antiarrhythmics) were not uniformly available, and their potential contribution could not be analyzed. This represents an important source of possible confounding.

The modest sample size also increases the risk of underpowering and selection bias, as many potentially eligible cases were excluded due to incomplete data. Restricting the cohort to patients with psychiatric consultation may limit generalizability, as other elderly patients on psychotropics presenting for medical rather than psychiatric reasons were not included.

Despite these limitations, we believe that the study offers meaningful insights into the cardiometabolic safety of psychotropic medications in a high-risk geriatric population. It underscores the need for integrated, multidisciplinary assessment at the interface of emergency medicine, psychiatry, and cardiology.

## 5. Conclusions

The present study sheds light on a clinically relevant but often overlooked intersection between psychotropic pharmacotherapy and cardiovascular risk in older adults. In a cohort of elderly patients evaluated in the Emergency Department for psychiatric symptoms, we observed a substantial prevalence of electrocardiographic abnormalities, particularly QTc prolongation, that was significantly associated with the use of psychotropic medications.

These findings emphasize the need for greater awareness among clinicians (psychiatrists, emergency physicians, and cardiologists alike) regarding the arrhythmogenic potential of certain drugs commonly prescribed to older patients, especially in acute care settings [51,52]. While psychotropic medications remain indispensable for managing agitation, mood disorders, and psychosis in the elderly, their use should be carefully balanced against cardiovascular safety concerns. This involves individualized risk assessment, ECG monitoring when appropriate, and consideration of safer pharmacological alternatives where feasible.

Importantly, our data also highlights that pharmacological risk is only one part of the equation. Non-modifiable factors such as advanced age and sex, as well as modifiable contributors like electrolyte disturbances, comorbidities, and polypharmacy, appear to play a key role in determining a patient’s overall vulnerability to repolarization abnormalities [53]. Addressing these broader determinants is as crucial as evaluating the medication profile itself.

Despite the limitations inherent to a retrospective and relatively small single-center study, we believe that these real-world observations provide valuable insights and raise pertinent clinical questions. In particular, they support the need for prospective, interdisciplinary research that can guide safer prescribing practices and develop practical tools for ECG risk stratification in frail geriatric patients.

In conclusion, a more integrated approach to the medical-psychiatric interface, one that transcends specialty boundaries, appears essential for optimizing both mental health outcomes and cardiac safety in the growing population of elderly patients.

## Figures and Tables

**Table 1:** Clinical and Therapeutic Characteristics by Sex.

Variable	All Patients (*n* = 77)	Male (*n* = 29)	Female (*n* = 48)	*p*-Value
Age (years, median [IQR])	72 [67–84]	72 [67–84]	74 [66–88]	0.411
EMS transport (%)	36.4	31	39.6	0.450
Triage code: Green (%)	19.5	20.7	18.7	
Triage code: Blue (%)	28.6	31	27.1	0.966
Triage code: Orange (%)	37.7	34.5	39.6	
Triage code: Red (%)	14.3	13.8	14.6	
Altered consciousness (%)	31.2	34.5	29.2	0.626
Inappropriate drug use (%)	13.0	13.8	12.5	0.870

Note: Values are expressed as medians [interquartile range] for continuous variables and percentages for categorical variables. *p*-values refer to comparisons between male and female subgroups. EMS: Emergency Medical Services.

**Table 2:** Multivariate Logistic Regression Predicting ECG Abnormalities.

Variable	Odds Ratio (OR)	95% Confidence Interval	*p*-Value
Psychotropic drug use	2.84	1.01–7.98	0.049
Age (per 1-year increase)	1.03	0.96–1.10	0.311
Female sex	0.89	0.36–2.21	0.801
Pre-existing cardiovascular disease	1.45	0.58–3.61	0.421

Note: ECG abnormalities include QTc prolongation (>450 ms in males, >470 ms in females) and/or QRS prolongation (≥120 ms). Odds ratios adjusted for all listed variables.

## Data Availability

The data supporting the findings of this study are available from the corresponding author upon reasonable request. However, restrictions apply to the availability of these data due to patient privacy and institutional regulations, and they are therefore not publicly available.
